# Long-term evaluation of patients with BMI = 50kg/m^2^ who underwent Bariatric Surgery

**DOI:** 10.1590/0100-6991e-20233397-en

**Published:** 2023-04-12

**Authors:** MATHEUS DUARTE MEIRA, FERNANDO DE SANTA CRUZ OLIVEIRA, LUCAS RIBEIRO COUTINHO, LUÍS HENRIQUE DE ALBUQUERQUE LEÃO, GÉSSICA DE PAULA VASCONCELOS, LUCIANA TEIXEIRA DE SIQUEIRA, ÁLVARO ANTÔNIO BANDEIRA FERRAZ

**Affiliations:** 1 - Universidade Federal de Pernambuco (UFPE), Pós-graduação em Cirurgia - Recife - PE - Brasil; 2 - Hospital dos Servidores do Estado (HSE), Serviço de Cirurgia Geral - Recife - PE - Brasil; 3 - Universidade Federal de Pernambuco (UFPE), Curso de Medicina - Recife - PE - Brasi; 4 - Hospital das Clínicas da Universidade Federal de Pernambuco (HCUFPE), Serviço de Cirurgia Geral - Recife - PE - Brasil; 5 - Universidade Federal de Pernambuco (UFPE), Departamento de Cirurgia - Recife - PE - Brasil

**Keywords:** Bariatric Surgery, Obesity, Morbid, Postoperative Complications, Cirurgia Bariátrica, Obesidade Mórbida, Complicações Pós-Operatórias

## Abstract

**Purpose:**

to determine the risks and benefits of bariatric surgery in patients with super obesity (SO) in comparison with obesity grades II and III.

**Methods:**

retrospective cohort that included a study group of 178 patients with SO and a control group of 181 patients with BMI 35-49.9Kg/m^2^. The groups were formed in a 1:1 nearest neighbor matching. The main variables were pre- and postoperative BMI and comorbidities, occurrence of severe postoperative complications, bowel obstruction, marginal ulcer, fistulae and 30-day death, besides the necessity of emergency room (ER) admission and abdominal computed tomography (CT) scans in the postoperative period due to acute abdomen.

**Results:**

the study group comprised 74.0% of women while the control group had 56.7%. The mean follow-up time was similar between both groups (5.48 x 6.09 years, p=0.216). There was no statistically significant difference on the prevalence of hypertension and T2D between the groups according to the surgical technique. All deaths occurred in the Study group (BMI = 50kg/m^2^) who underwent RYGB. There was no difference between the groups regarding the occurrence of severe complications. Data on ER admissions and the need for abdominal CT to investigate postoperative abdominal pain did not show statistically significant difference between the groups.

**Conclusion:**

despite the high risk related to bariatric surgery in patients with SO, the benefits related to the remission of comorbidities are significant; although being lower than those found in patients with milder grades of obesity.

## RESUMO

**Objetivo:**

determinar os riscos e benefícios da cirurgia bariátrica em pacientes com superobesidade em comparação com obesidade graus II e III.

**Métodos:**

coorte retrospectiva que incluiu um grupo Estudo com 178 pacientes portadores de superobesidade e um controle com 181 pacientes com IMC 35-49.9Kg/m^2^. Os grupos foram formados numa razão 1:1 com pareamento “vizinho próximo”. As principais variáveis foram comorbidades e IMC pré e pós-operatório, ocorrência de complicações pós-operatórias severas, obstrução intestinal, úlcera marginal, fístula e morte em 30 dias, além da necessidade de idas à emergência e realização de tomografia computadorizada (CT) para investigação de abdome agudo.

**Resultados:**

o grupo Estudo foi composto por 74.0% de mulheres, enquanto o Controle teve apenas 56.7%. O tempo médio de seguimento foi similar em ambos os grupos (5.48 x 6.09 anos, p=0.216). Não houve diferença estatisticamente significativa em relação à prevalência de diabetes entre os grupos nem entre a técnica cirúrgica empregada. Todos os óbitos ocorreram no grupo Estudo (IMC = 50kg/m^2^), com apenas pacientes submetidos ao BGYR. Não houve diferença significativa entre os grupos em relação à ocorrência de complicações pós-operatórias. Ao avaliar idas à emergência e necessidade de TC para investigar dor abdominal, também não foi observada difrença significativa entre os grupos.

**Conclusão:**

apesar do alto risco cirúrgico implicado em pacientes portadores de superobesidade, os benefícios relacionados à remissão de comorbidades ainda são significativos; contudo os resultados parecem ser inferiores aos pacientes com graus mais leves de obesidade.

## INTRODUCTION

The obesity pandemic figures as one of the most urgent and challenging health issues to be faced in the current century due to increasing prevalence rates^1^ ^,^ ^2^. Interestingly and discouragingly, the severe form of obesity (BMI ≥50kg/m^2^) present even worse numbers, increasing 2-3 times faster than the incidence of class I obesity (BMI 30-34.9kg/m^2^) in the USA^2^ ^,^ ^3^.

Bariatric surgery consists of the most effective approach to treat obesity in the short- and long-terms, leading to adequate weight loss and resolution of comorbidities^4^. However, when it comes to patients with BMI ≥50Kg/m^2^, several issues must be taken into consideration before indicating this procedure^5^ ^,^ ^6^. The first point that must be discussed with the patient is related to the technical difficulty of carrying out this procedure in the referred population, since such high BMIs are related to increased liver size and visceral fat^7^. In addition, this technical challenge might be responsible for a significantly longer operating time and a larger number of pre- and post-operative complications when compared with operations in patients with lower BMIs^8^ ^,^ ^9^. Second, studies have shown that bariatric surgical procedures are less effective in these patients, as they had lower odds of achieving adequate weight loss and higher odds of poorly controlling comorbidities^10^ ^,^ ^11^.

Given the life-threatening scenario that a BMI ≥50kg/m^2^ represents, it is of paramount necessity that studies are developed in order to elucidate questions regarding the balance between the risks and benefits of bariatric surgery in this population. Therefore, this paper presents a long-term follow-up of patients with BMI ≥50kg/m^2^ who underwent bariatric surgery. 

## METHODS

### Study design

This is a retrospective cohort study conducted in our center with patients who underwent RYGB or sleeve gastrectomy (SG) between 2005 and 2018. The study group comprised 178 patients with BMI ≥50Kg/m^2^ (SO), while the control group comprised 181 patients with BMI 35-49.9Kg/m^2^ (obesity grades II and III). All patients aged ≥18 years, of both genders, with BMI ≥35kg/m^2^ were candidates for the study protocol. Candidates for revisional bariatric surgery were excluded from the analysis. Data were gathered in an electronic database.

The following variables were studied: 30-day postoperative mortality, resolution of comorbidities, cholelithiasis, number of emergency room (ER) admissions, necessity of abdominal computed tomography (CT) to investigate acute abdomen in the ER, and surgical complications, including gastrointestinal fistula, anastomotic leak, bowel obstruction, and marginal ulcer. Severe complications were defined as any postoperative complication that required surgical or endoscopic management (Clavien-Dindo ≥III)^12^. The Clavien-Dindo classification was used in order to simplify the stratification of the data on surgical complications.

### Group formation

The groups were formed in a 1:1 nearest neighbor matching. We included all patients with BMI ≥50Kg/m^2^ who underwent RYGB or SG within the study period in our center to integrate the study group (SO). A control group (obesity grades II and III) was formed by selecting the first patient with BMI below 50Kg/m^2^ who was operated after the operation of an integrant of the study group (nearest neighbor matching). This strategy of group formation was implemented to reduce the risk of selection bias.

### Surgical Procedures

RYGB were performed with a 50ml gastric pouch, without ring, and the lengths of the alimentary and biliopancreatic limbs were 150cm and 100cm, respectively. All patients had a higher than 250-cm-long common limb.

For SG, the stapler (60mm cartridge) is placed parallel to a 36 Fr Fouchet bougie inserted into the stomach. After complete stapling, a transmural continuous suture line is performed with 3-0 PDS^®^ along the stapling line.

### Statistical analysis

A Microsoft Excel spreadsheet was created to analyze the data, which was transferred to the SPSS software, version 18, to perform data analysis. Then, the percentage frequencies of the variables were calculated, and the frequency distributions were determined to evaluate the demographic profile of the patients in this study. Mean and standard deviation were calculated for the quantitative variables. The Kolmogorov-Smirnov test was applied in order to evaluate normal distribution. In cases in which normality was confirmed, the Student’s t-test for paired samples was applied in order to compare the variables between each moment of evaluation. If the normality hypothesis was refuted, the Wilcoxon test was applied. All conclusions were made taking into consideration the significance level of 5% (p-value <0.05).

## RESULTS

Table 1 shows the results of the demographic profile of the sample studied. The statistical analysis shows that the Study group (SO) presented the lowest mean age and the highest prevalence of hypertension at baseline. The mean follow-up time was similar between both groups.

**Table 1:** Demographic profile of the patients studied.

	Group		
Variable	Study group	Control group	p-value
Total: n (%)	178 (100.0)	181 (100.0)	
Age: Mean ± SD	37.67 ± 12.17	44.08 ± 12.50	p^1^<0.001*
Gender: n (%)			
Male	77 (43.3)	47 (26.0)	p^2^=0.001*
Female	101 (56.7)	134 (74.0)	
Hypertension-pre: n (%)	123 (69.1)	107 (59.1)	p^2^=0.049*
T2D-pre: n (%)	41 (23.0)	59 (32.6)	p^2^=0.043*
BMI-pre: Mean ± SD	53.84 ± 3.49	41.66 ± 4.47	
Follow-up: Mean ± SD	5.48 ± 4.62	6.09 ± 4.63	p^1^=0.216
Median (P25; P75)	4.00 (1.25; 9.00)	5,00 (2.00; 9.00)	

*Significant difference at the level of 5.0%; ^1^By t-Student test with equal variances; ^2^Pearson’s chi-square test.

Table 2 shows the comparison of comorbidities between groups and according to the surgical technique implemented. The prevalence of GERD was higher among patients with a BMI <50kg/m^2^ (control group), both preoperatively and postoperatively. There was no statistically significant difference on the prevalence of hypertension and T2D between the groups according to the surgical technique.

**Table 2:** Evaluation of pre- and postoperative comorbidities according to the group and surgical technique.

		Group		
Surgical technique	Variable	Study group n (%)	Control group n (%)	p-value
	Hypertension pre			
RYGB	Yes	102 (71.8)	91 (63.2)	p^1^=0.119
	No	40 (28.2)	53 (36.8)	
SG	Yes	21 (58.3)	16 (43.2)	p^1^=0.197
	No	15 (41.7)	21 (56.8)	
	Hypertension post			
RYGB	Yes	55 (38.7)	43 (29.8)	p^1^=0.355
	No	87 (61.3)	101 (70.2)	
SG	Yes	7 (19.4)	7 (18.9)	p^1^=0.517
SG	No	29 (80.6)	30 (81.1)	
	T2D pre			
RYGB	Yes	35 (24.6)	48 (33.3)	p^1^=0.106
	No	107 (75.4)	96 (66.7)	
RYGB	Yes	6 (16.7)	11 (29.7)	p^1^=0.187
SG	No	30 (83.3)	26 (70.3)	
		Group		
Surgical technique	Variable	Study group n (%)	Control group n (%)	p-value
	T2D post			
RYGB	Yes	9 (6.3)	16 (11,1)	p^1^=0.455
	No	133 (93.7)	128(88.9)	
SG	Yes	-	3 (8.1)	p^2^=0.515
	No	36 (100.0)	34 (91.9)	
	GERD pre			
RYGB	Yes	31 (21.8)	50 (34.7)	p=0.015*
	No	111 (78.2)	94 (65.3)	
SG	Yes	6 (16.7)	11 (29.7)	p=0.186
	No	30 (83.3)	26 (70.3)	
	GERD post			
RYGB	Yes	4 (2.8)	20 (13.8)	p<0.01*
	No	138 (97.2)	124 (86.2)	
SG	Yes	2 (5.5)	1 (2.7)	p=0.539
	No	34 (94.5)	36 (97.3)	

*Significant difference at the level of 5.0%; ^1^Pearson’s chi-square test for comparison between groups; ^2^Fisher’s exact test for comparison between groups.

Table 3 shows the occurrence of adverse events in the postoperative period. All deaths (n=3) occurred in the Study group (BMI ≥50kg/m^2^) who underwent RYGB. There was no difference between the groups or between the surgical technique regarding the occurrence of severe complications (Clavien-Dindo ≥III). The incidence of anemia was significantly higher in the control group (p=0.017) and in those patients who underwent RYGB (p=0.031). 

**Table 3:** Adverse events by group and surgical technique.

		Group		
Surgical technique	Variable	Study group n (%)	Control group n (%)	p-value
BGYR	Severe complications	8 (5,63)	7 (4,86)	p^1^ =0,769
GV		2 (5,88)	1 (2 .7)	p^1^ =0,505
p-valor		p^3^ =0,955	p^3^ =0,299	
BGYR	Cholelithiasis	22 (15,5)	20 (13,9)	p^1^ =0,702
GV		8 (22.2)	7 (18,9)	p^1^ =0,727
p-valor		p^3^ =0,335	p^3^ =0,444	
BGYR	Anemia	17 (12,0)	24 (16,7)	
GV		2 (5,6)	1 (2,7)	p^3^ =0,017*
p-valor		p^4^ =0,372	p^3^ =0,031*	
BGYR	Bowel obstruction	4 (2,8)	6 (4.2)	p^1^ =0,750
GV		-	-	**
p-valor		p^3^ =0,584	p^3^ =0,349	
BGYR	Marginal ulcer	3 (2.1)	3 (2.1)	p^1^ =1,000
GV		2 (5,6)	1 (2,7)	p^1^ =0,615
p-valor		p^3^ =0,266	p^3^ =1,000	
BGYR	Úlcera marginal	6 (4.2)	3 (2.1)	p^1^ =0,333
GV		-	-	**
p-valor		**	**	
		Group		
Surgical technique	Variable	Study group n (%)	Control group n (%)	p-value
	Death (up to 30 days)	3 (2.1)		p^1^ =0,023*
BGYR	Anastomotic leak	1 (0,7)	-	
	Pulmonary thromboembolism (PTE)	1 (0,7)	-	
	Cardiovascular	1 (0,7)	-	

*Significant difference at the level of 5.0%; ^1^Pearson’s chi-square test for comparison between groups; ^2^Fisher’s exact test for comparison between groups; ^3^Pearson’s chi-square test to compare surgical techniques.

Table 4 shows data on emergency room admissions and the need for abdominal CT to investigate postoperative abdominal pain. There was no statistically significant difference between the groups. 

**Table 4:** Emergency room admissions and necessity of abdominal CT.

		Grupos		
Variable	Technique	Study group n (%)	Control group n (%)	p-value
ER admissions	RYGB	23 (16.2)	22 (15.3)	p^1^=0.831
	SG	8 (22.2)	4 (10.8)	p^1^=0.188
Abdominal CT	RYGB	8 (5.6)	15 (10.4)	p^1^=0.137
	SG	2 (5.6)	2 (5.4)	p^1^=1.00

^1^Pearson chi-square test.

## DISCUSSION

Bariatric surgery is an intervention aimed at weight loss and regression of comorbidities associated with obesity in any of its stages^13^. Thus, although RYGB and SG are the most performed surgical techniques in the world, the results in the literature are conflicting when these techniques are evaluated for a specific group of patients, the SO. Presenting a higher incidence of postoperative complications associated with a higher surgical risk due to a larger number of comorbidities and the technical difficulty to perform the surgery, there are few studies evaluating the follow-up of SO patients and comparing the aforementioned surgical techniques^14^.

The reduction in BMI is an important factor to evaluate the success of bariatric surgery and the postoperative follow-up of patients. Greater postoperative percentage of excess weight loss is expected from those with higher BMIs^15^. However, the evaluative parameters for the reduction of BMI in the SO population remain conflicting, as a treatment failure of 44% was observed in a group of SO patients submitted to SG when BMI <40kg/m^2^ or BMI <35kg/m^2^ associated with reduction of comorbidities were used as criteria for surgical success16. On the other hand, the treatment failure rate drops to 22% when a 50% excess weight loss is taken into consideration for the success criteria. Regardless of the surgical technique, the SO patients analyzed in this study presented a BMI <40kg/m^2^ until the fifth year of follow-up. This data remained stable for patients undergoing RYGB and followed-up for more than 5-10 years. 

The first two years after bariatric surgery represent the period when patients lose more weight. When comparing the two techniques, weight loss in patients undergoing RYGB is greater up to 24 months^17^ ^-^ ^19^. However, we did not find any statistical difference between the surgical techniques in the weight loss curve of SO patients in our sample. A limitation of these findings is that the number of patients who underwent RYGB was considerably higher than the number of patients in the postoperative period of SG.

In association with the reduction in BMI, other important parameters to be analyzed in the postoperative period of bariatric surgery are the control or resolution of comorbidities. SO patients present comorbidities such as T2D, hypertension, heart failure, and chronic obstructive pulmonary disease at higher stages of complexity when compared to patients with lower grades of obesity, which increases the surgical risk and determines a challenging perioperative management^20^ ^-^ ^22^. Sutter et al. showed that the postoperative improvement in obesity-related comorbidities is similar between SO and non-SO patients^23^. They also observed a direct relationship between remission of dyslipidemia and T2D and the percentage of excess weight loss, achieving equivalent remission rates between their groups. In the present study, patients with BMI 35-49.9Kg/m^2^ presented a higher rate of hypertensin remission (53.3% vs. 42.4%), while the group of SO patients presented a higher rate of T2D remission (84.1% vs. 65.6%). Surgical techniques did not differ statistically. 

In addition to the postoperative analysis of hypertension and T2D, another important comorbidity to be evaluated is GERD. The benefit of RYGB in GERD is evident in the literature. However, there are still doubts on the behavior of this disease in patients with pre-existing GERD, besides questions regarding the possibility of a greater occurrence of GERD after this surgery^24^. In our study, clinical complaints related to GERD decreased for both groups regardless of the surgical technique, and the postoperative prevalence of the clinical condition was lower in the group of SO patients submitted to RYGB. Thus, our findings reinforced the indication of RYGB in patients with pre-existing GERD.

Concomitantly with the analysis of the postoperative clinical outcomes of bariatric surgery in the SO population, the occurrence of short and long-term postoperative complications is one of the central issues regarding the safety and surgical feasibility of this procedure in this population. In this context, the analysis of 356,621 patients with obesity or SO showed that the groups with higher BMI maintained a low mortality rate in the first 30 days after surgery (0.33%)^25^. Similarly, a meta-analysis performed by Wang et al. based on 12 retrospective studies showed perioperative mortality and 30-day mortality rates of 0.4% and 0.3% in patients with SO undergoing RYGB (n=42,631) and SG (n=9,415), respectively^18^. 

In our study, the sample also suggested a trend towards greater surgical morbidity and mortality in patients with SO undergoing RYGB. The deaths within the immediate postoperative period were exclusive to the SO group, with a statistical difference in relation to the group of patients with obesity (2.1% vs. 0%; p=0.023). On the other hand, it is important to highlight that the rate of the present study was considerably higher when compared to the aforementioned references. This fact may be related to the smaller number of patients included in the analysis and to the process of improving preoperative preparation, since the individuals included in the RYGB group underwent the procedure since 2005.

As stated before in this paper, individuals with SO tend to present more negative postoperative outcomes; however, there is still no clear consensus regarding the occurrence of serious complications, which are defined in the present analysis as grade III Clavien-Dindo classification. Despite the larger absolute number of serious complications in the SO group compared to the study group (5.62% vs. 4.42%) found in this study, there was no statistically significant difference between these groups for this parameter. The main and most feared causes of surgical reoperation in patients undergoing bariatric surgery are anastomotic leaks, which imply the need for a surgical intervention in up to 89% of cases as described by Smith et al.^26^. Alizadeh et al. retrospectively studied 133,478 patients who underwent SG (n=92,495) and RYGB (n=40,983) and found an overall anastomotic leak rate of 0.7%^27^. Our study showed an overall fistula rate of 2.8% in the SO group, as well as a higher percentage in both the RYGB (2.1% vs 1.2%) and the SG group (5.6% vs 0.5%). We emphasize that the influence of the higher baseline BMI in the current study (53.8Kg/m^2^) may be responsible for the higher rates of fistula in comparison with the standard literature. 

The dehiscence rate of the patients analyzed in our study corroborated data from the systematic review (n=40,653) conducted by Gagner et al., who described a fistula incidence of up to 2.7%^28^. In addition, from a comparison between the two groups in our analysis, there was no statistical significance for the slightly higher frequency of the SO group (2.8% vs 2.2%), which seems to corroborate the safety of bariatric surgery in patients with a BMI ≥50kg/m ^2^. 

The present study has several limitations. First, related to the very small number of patients submitted to SG. This discrepancy weakens the analysis of the surgical techniques. Second, the retrospective and observational nature of the paper hinders any definitive conclusion regarding the theme under scope. Third, the follow-up time was very heterogenous within each group, turning it difficult to establish a reliable correlation in the timeline.

Based on the current findings aside with the literature data, it is possible to trace strategies aiming to achieve the best benefits with the lowest risks in patients with BMI ≥50kg/m^2^ undergoing bariatric surgery. We believe that the routine use of intragastric balloons and the proposal of a two-steps intervention may be the key. A two-step intervention would provide a smaller operation in the first place (e.g. SG) and a definitive procedure in the second place, performing a conversion to RYGB, SADI-S or intestinal transit bipartition when the patient have already achieved important weight loss and comorbidity control.

## CONCLUSION

Patients with BMI ≥50kg/m^2^ present higher incidence of surgical complications and postoperative death when compared with patients with obesity grade ≤III. Despite this high risk, our findings indicate that the benefits related to the remission of comorbidities are significant, although still being lower than those found in patients with a BMI <50kg/m^2^. Regarding the risks of the procedure, RYGB seems to be associated with a higher incidence of complications in the postoperative period, including death within 30 days. Large clinical trials and robust meta-analyses are still needed to confirm this hypothesis raised in the present observational study.

### Ethical approval:

All procedures performed in this study involving human participants were in accordance with the ethical standards of the institutional research committee and the 1964 Helsinki declaration and its later amendments, or comparable ethical standards. This research project was approved by the Ethics Committee of our institution under the protocol CAAE no. 50097321.2.0000.8807. For this type of study formal consent is not required.
